# Synergistic Effect of Proteinase Activity by Purification and Identification of Toxic Protease From *Nemopilema nomurai*

**DOI:** 10.3389/fphar.2021.791847

**Published:** 2021-11-25

**Authors:** Chunlin Yu, Rongfeng Li, Xiujing Yin, Huahua Yu, Pengcheng Li

**Affiliations:** ^1^ CAS and Shandong Province Key Laboratory of Experimental Marine Biology, Center for Ocean Mega-Science, Institute of Oceanology, Chinese Academy of Sciences, Qingdao, Qingdao, China; ^2^ College of Earth and Planetary Sciences, University of Chinese Academy of Sciences, Beijing, China; ^3^ Laboratory for Marine Drugs and Bioproducts, Pilot National Laboratory for Marine Science and Technology, Qingdao, China

**Keywords:** jellyfish *Nemopilema nomurai*, purification, protease, metalloprotease, synergistic effect

## Abstract

Scyphozoan *Nemopilema nomurai* envenomation is an unresolved threat to human health in Asian waters. *Nemopilema nomurai* venom metalloproteinases show important toxicities in skin damage and inflammation, but there is still no purified protein for further studies. In this study, high proteinase activity fractions in tentacle autolysis were isolated by ammonium sulfate precipitation, DEAE Sepharose Fast Flow, and Superdex 75 chromatography successively. Purification was guided by azocasein hydrolysis activity and SDS-PAGE. The final products were analyzed by LC-MS/MS. Four elution peaks purified by Superdex 75 chromatography had multiple protein bands but did not show proteinase activity. These fractions would recover proteinase activity after mixing again. Regulation mechanisms were speculated as binding metalloproteinase regulator or disaggregating metalloproteinase inhibitor by LC-MS/MS analysis. For the first time, a synergistic effect in *N. nomurai* proteinase activity was found in the purification process.

## Introduction

Jellyfish sting is an important health problem for persons engaged in marine activities, and the symptoms of victims range from itch, pain, inflammation, and edema to cutaneous necrosis and even death (Cegolon et al., 2013). Venom from jellyfish *Nemopilema nomurai* is responsible for the symptoms of jellyfish envenomation. *N. nomurai* is widely distributed in the Yellow Sea and East China Sea every summer season (Sun et al., 2015a; Sun et al., 2015b). It has been regarded as a synonymy of *Stomolophus meleagris* in some research literatures (Liumin et al., 2011). In recent decades, blooms of *N. nomurai* have been of frequent occurrence and became more of a threat to humans in Chinese, Japanese, and Korean waters (Kawahara et al., 2006; Liumin et al., 2011; Xu et al., 2013; Yoon et al., 2014; Sun et al., 2015a; Sun et al., 2015b). This makes *N. nomurai* venom research more necessary and important.

Jellyfish *N. nomurai* venom is a complex mixture of peptides and proteins (Li et al., 2014) and has a variety of bioactivities such as hemolytic and antioxidant activities (Yu et al., 2005; Li et al., 2012; Li et al., 2013; Yue et al., 2021). According to the proteome and transcriptome analyses, metalloproteinases are main components in *N. nomurai* venom (Li et al., 2014). The crude venom extracted from nematocysts was identified to have significant metalloproteinase activity (Yue et al., 2017b) and played an important role in pro-inflammatory activity, edematogenic effects, lethality, and cytotoxicity (Lee et al., 2011; Yue et al., 2017b; Li et al., 2018; Yue et al., 2021). Through chromatography, some *N. nomurai* toxins were partially purified, such as hemolytic toxin SmTX (Li et al., 2013) and lethal toxin NnLF (Li et al., 2020). Protease in *N. nomurai* venom, isolated and identified by zymogram, was identified to contain many other protein components (Yue et al., 2017a). A purified protease with only one SDS-PAGE band was isolated from *N. nomurai* venom by HiPrep 26/60 Sephacryl S-200 column, which did not match any high similarity protease in LC-MS/MS analysis (Yue et al., 2021).

Several protein toxins were successfully purified from jellyfish venom by chromatography, such as cytotoxin CcTX-1 and antioxidant protein SmP90 (Rottini et al., 1995; Yang et al., 2003; Helmholz et al., 2008; Lassen et al., 2011; Lassen et al., 2012; Li et al., 2012; Horiike et al., 2015). But more toxins from jellyfish were partially purified (Bloom et al., 1998; Chung et al., 2001; Sanchez-Rodriguez et al., 2006; Li et al., 2011; Li et al., 2013; Li et al., 2018; Li et al., 2020). For example, Rastogi et al. (2017) identified a 95 kDa metalloproteinase in a partially purified product of *Rhizostoma pulmo* (barrel jellyfish) venom. But there are still no single metalloproteinase purified from the jellyfish venom, which limits the analysis of its molecular structure and action mechanism. In addition to the small amount of toxin and instability, synergistic effects of toxins may increase the difficulty of single metalloproteinase purification.

Synergistic effects in snake venom have been demonstrated to occur between different toxins with different patterns owing to the amounts of toxin purification, characterization, and pathological researches (Doley and Kini, 2009; Xiong and Huang, 2018). The synergetic pain activation mechanism of scorpion toxin BmP01 was deeply revealed based on its molecular structure researches (Yang et al., 2017). Metalloproteinases in snake venom showed different interactions to other subunits and components (Doley and Kini, 2009; Xiong and Huang, 2018). However, there is no report on the synergistic effects of jellyfish toxins, and the regulation mechanism of jellyfish venom metalloproteinase has not been revealed.

In the present study, the toxic protease components in *N. nomurai* tentacle tissue autolysis were purified by activity-guided chromatography. Protein components of the final purified products, which showed a synergistic effect of proteinase activity, were identified by LC-MS/MS analysis to explore the possible active components and regulation mechanism. This study may provide references for further research on the NnVMP and therapy of jellyfish stings.

## Materials and Methods

### Venomous Sample Collection

The venomous sample used in protease purification was collected from the supernatant of *N. nomurai* tentacle autolysis. *N. nomurai* was collected from the coastal waters of Huangshan Village, in the Yellow Sea, on August 29, 2018. Tentacle tissues were cut off from *N. nomurai* and immediately transported back to the laboratory in an ice bath. Every package of the samples was a mixture of multiple *N. nomurai* individuals. The tentacle tissues were mixed with 50% (v/v) precooled filtered natural seawater and autolyzed at 4°C for 3 days. Then, autolysis solutions were centrifuged at 3000 *g* for 15 min. The supernatant was collected as venomous samples for protease purification. Protein concentrations were determined using FolinCiocalteu's phenol reagent (Dingguo Changsheng Biotechnology Co. Ltd., Beijing, China) according to the manufacturer's instructions.

### Ammonium Sulfate Fractional Precipitation

Ammonium sulfate fractional precipitation was performed in a 0°C chromatography freezer with sustained magnetic stirring. An appropriate amount of solid ammonium sulfate was added in small amounts repeatedly to 1 L of the venomous samples and stirred overnight in a 0°C freezer. Then, the sample solutions were centrifuged at 10000 *g*, at 4°C for 5 min. All of the supernatants were collected for the preparation of protein precipitation in the subsequent ammonium sulfate saturation. The protein precipitation was redissolved in 100 ml PBS (20 mM, pH 7.4) and dialyzed in the same PBS solution at 4°C for 48 h by Spectra/Por CE dialysis tubing, 500–1000 MWCO. Then, the sample solutions were centrifuged at 4°C, 10000 *g* for 5 min and the supernatant collected for further experiment. The ammonium sulfate saturations set in this experiment were 20, 30, 40, 50, 60, 70, and 80%.

### Chromatography Purification

About 30 mg protein precipitation collected from 80% ammonium sulfate saturation was used in DEAE Sepharose Fast Flow chromatography. First, the sample solutions were concentrated by Millipore concentrators, 3,000 Da MWCO, at 4°C, 6000 g, and filtered with a 22 μm membrane. The protein samples were purified with a Hiprep DEAE FF 16/10 chromatography column (GE Healthcare, Princeton, NJ) that was coupled to a fast protein liquid AKTA pure chromatography system. The proteins were eluted by a discontinuous NaCl gradient (0.1–2 M), 3 ml/min. The equilibration buffer was 20 mM PBS. Elution buffer A was 20 mM PBS with 2 M NaCl, pH 7.4. Each elution peak was pooled and concentrated to test SDS-PAGE and metalloprotease activity. The concentrated fraction of 0.2 M NaCl elution peak was then filtered and submitted to a HiLoad 16/60 Superdex 75 column (GE Healthcare) with elution buffer B (0.15 M NaCl, 20 mM PBS, pH 7.4), 1 ml/min. Each elution peak was pooled, concentrated, and tested in SDS-PAGE for metalloprotease activity. The whole chromatography system worked in a 4°C freezer.

### SDS-PAGE

SDS-PAGE was performed according to Laemmli's method (Laemmli, 1970). Briefly, 20 μg of the protein sample mixed with nonreducing 5× Protein Loading Buffer (Nanjing Jiancheng Bioengineering Institute, Nanjing, China) was incubated for 5 min at 100°C and then loaded into a 12% SurePAGE precast gel (GenScript, New Jersey, United States). Protein samples were separated in Tris-MOPS-SDS running buffer (GenScript, New Jersey, United States) in a Bio-Rad Mini-PROTEAN Tetra System (Bio-Rad, California, United States) at 120 V for approximately 90 min. Gels were stained with 0.5% Coomassie brilliant blue R-250 and then photographed and analyzed by a Bio-Rad Gel Doc EZ Imager (Bio-Rad, California, United States). The low molecular standard (Yuanye, Shanghai, China) includes rabbit phosphorylase b, 97.4 kDa; bovine serum albumin, 66.2 kDa; rabbit actin, 43.0 kDa; bovine carbonic anhydrase, 31.0 kDa; trypsin inhibitor, 20.1 kDa; and hen egg-white lysozyme, 14.4 kDa.

### Proteinase Activity

Proteinase activity was detected using azocasein according to a previously reported method (Wang et al., 2004) with minor modifications. Briefly, 12.5 μg of protein sample was added to 100 μL of 5 mg/ml azocasein (in 50 mM Tris-HCl, 100 mM NaCl, 5 mM CaCl_2_, pH 8.8) in a 1.5 ml centrifuge tube and then incubated at 37°C for 90 min. The reaction was stopped by adding 200 μL of 5% trichloroacetic acid at room temperature for 30 min. After centrifugation at 10,000 *g* for 20 min, 150 μL of the supernatant was transferred to a 96-well plate and mixed with 150 μL of 0.5 M NaOH. The absorbance was measured by an Infinite M100 plate reader (Tecan Group Ltd., Männedorf, Switzerland) at 450 nm. The PBS group was set as the negative control. Proteinase activity was shown as U/mg.

### LC-MS/MS Identification and Analysis

The protein components identification of fractions C and D was conducted by LC-MS/MS detection and spectra analysis. Briefly, SDT buffer was added to the protein powder sample. The lysate was sonicated and then boiled for 15 min. After being centrifuged at 14000 *g* for 40 min, the supernatant was quantified with the BCA Protein Assay Kit (Bio-Rad, United States). Then, 20 µg of proteins from each sample were mixed with 5× loading buffer and boiled for 5 min. The proteins were separated on 12.5% SDS-PAGE. Protein bands were visualized by Coomassie blue R-250 staining. 50 μg of sample was added to UA buffer, and DTT and iodoacetamide were then added to reduce and block the cysteine residues. Then trypsin was added. The mixtures were all incubated at 37°C for 16–18 h. Each fraction was injected for nano LC-MS/MS analysis. The peptide mixture was loaded onto a reverse-phase trap column (Thermo Scientific Acclaim PepMap100, 100 μm × 2 cm, NanoViper C18) connected to the C18 reversed phase analytical column (Thermo Scientific Easy Column, 10 cm long, 75 μm inner diameter, 3 μm resin) in buffer A (0.1% formic acid) and separated with a linear gradient of buffer B (84% acetonitrile and 0.1% formic acid) at a flow rate of 300 nL/min controlled by IntelliFlow technology. LC-MS/MS analysis was performed on a Q Exactive mass spectrometer (Thermo Scientific) that was coupled to Easy-nLC (Proxeon Biosystems, now Thermo Fisher Scientific) for 30 min. The mass spectrometer was operated in the positive ion mode. MS data were acquired using a data-dependent top10 method dynamically choosing the most abundant precursor ions from the survey scan (300–1,800 m/z) for HCD fragmentation. Automatic gain control target was set to 3e6 and maximum inject time to 10 ms. Dynamic exclusion duration was 40.0 s. Survey scans were acquired at a resolution of 70,000 at m/z 200, and resolution for HCD spectra was set to 17,500 at m/z 200, and isolation width was 2 m/z. Normalized collision energy was 30 eV, and the underfill ratio, which specifies the minimum percentage of the target value likely to be reached at maximum fill time, was defined as 0.1%. The instrument was run with peptide recognition mode enabled. MS/MS spectra were searched in Tox-Prot (Jungo and Bairoch, 2005) (https://www.UniProt.org/program/Toxins) and *N. nomurai* genome database (Kim et al., 2019) (https://www.ncbi.nlm.nih.gov/genome/?term=Nemopilema+nomurai) with a mass tolerance for precursor ion of 20 ppm and MS/MS tolerance for 0.1 Da. Each identified protein should contain at least 1 unique peptide. Only ion scores >20 indicate identity or extensive homology (*p* < 0.05).

### Statistical Analysis

The results were expressed as the mean ± SEM. The significant differences in the mean between various experimental groups were analyzed by an analysis of variance, followed by Tukey's test. *p* < 0.05 was considered statistically significant.

## Results

### Isolation of High Proteinase Activity Fractions

**FIGURE 1:**
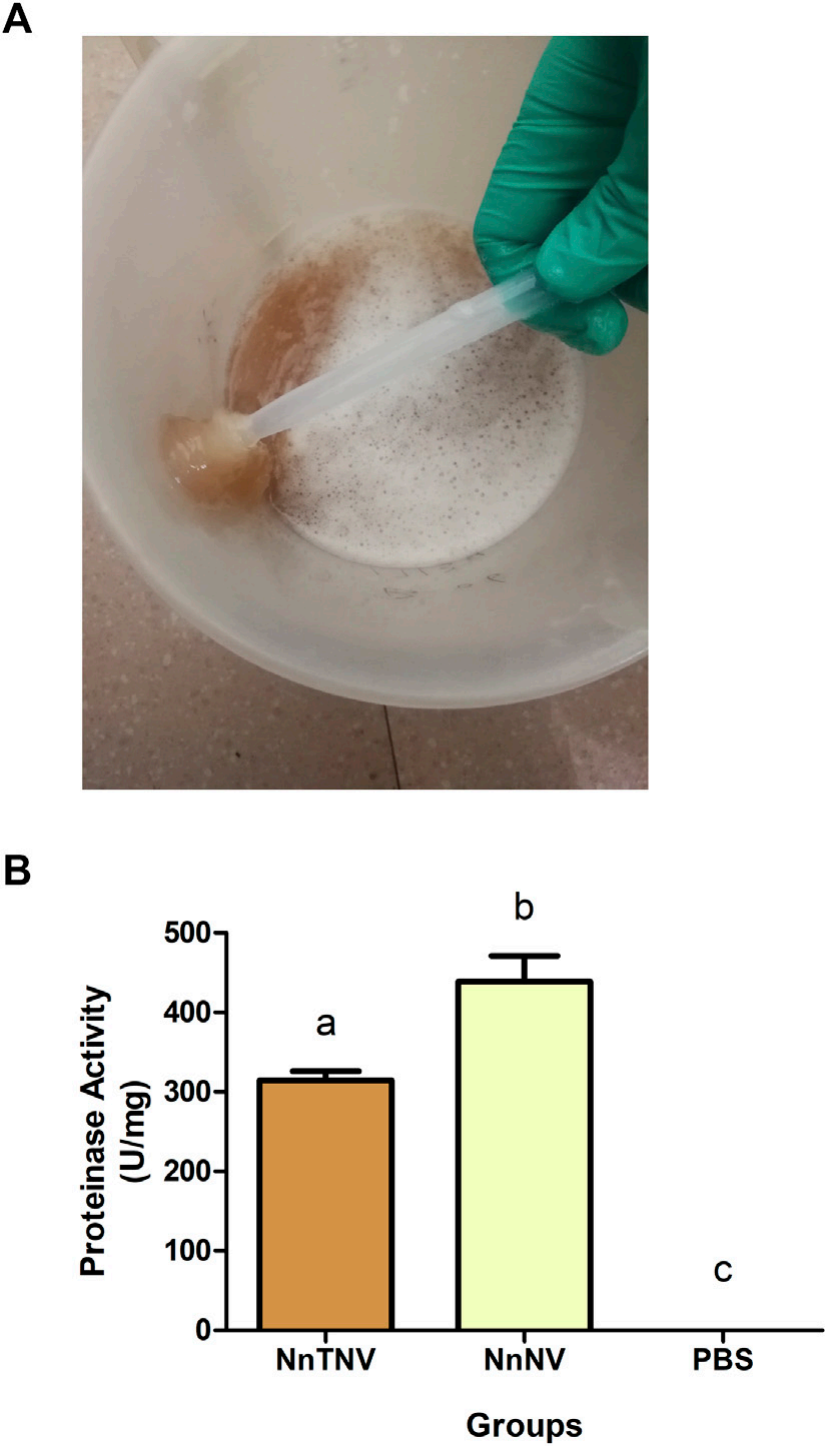
Venomous sample preparation and proteinase activity assay. **(A)** Fresh *Nemopilema nomurai* tentacle tissues mixed with 50% (v/v) filtered natural seawater and completely autolyzed in 3–5 days. Supernatant was used in further experiments. **(B)** Proteinase activity of venomous samples. NnTNV: *Nemopilema nomurai* tentacle autolysis; NnNV: *Nemopilema nomurai* nematocyst venom. Both venomous samples showed significant proteinase activity, *p* < 0.05. NnNV was 438.6 U/mg. NnTNV was 314.4 U/mg.

The venomous sample used in the purification process was the supernatant of *N. nomurai* tentacle autolysis (NnTNV, Figure 1A). In Cnidaria, toxins may also exist in other tissues than nematocysts (Zhang et al., 2003; Xiao et al., 2011). In addition, during the tentacle autolysis, some nematocysts were discharged. So, NnTNV contained tentacle and nematocyst samples and showed significant proteinase activity measured as 314 U/mg (Figure 1B). Moreover, the operation of extracting nematocysts venom, which has been described in a previous research (Li et al., 2011), reduced the protein content by filtration, centrifugation, and ultrasonication. So, NnTNV is more suitable for multistep purification because it has significant activity, more sample volume, and higher protein concentration as shown in Supplementary Table S1.

**FIGURE 2:**
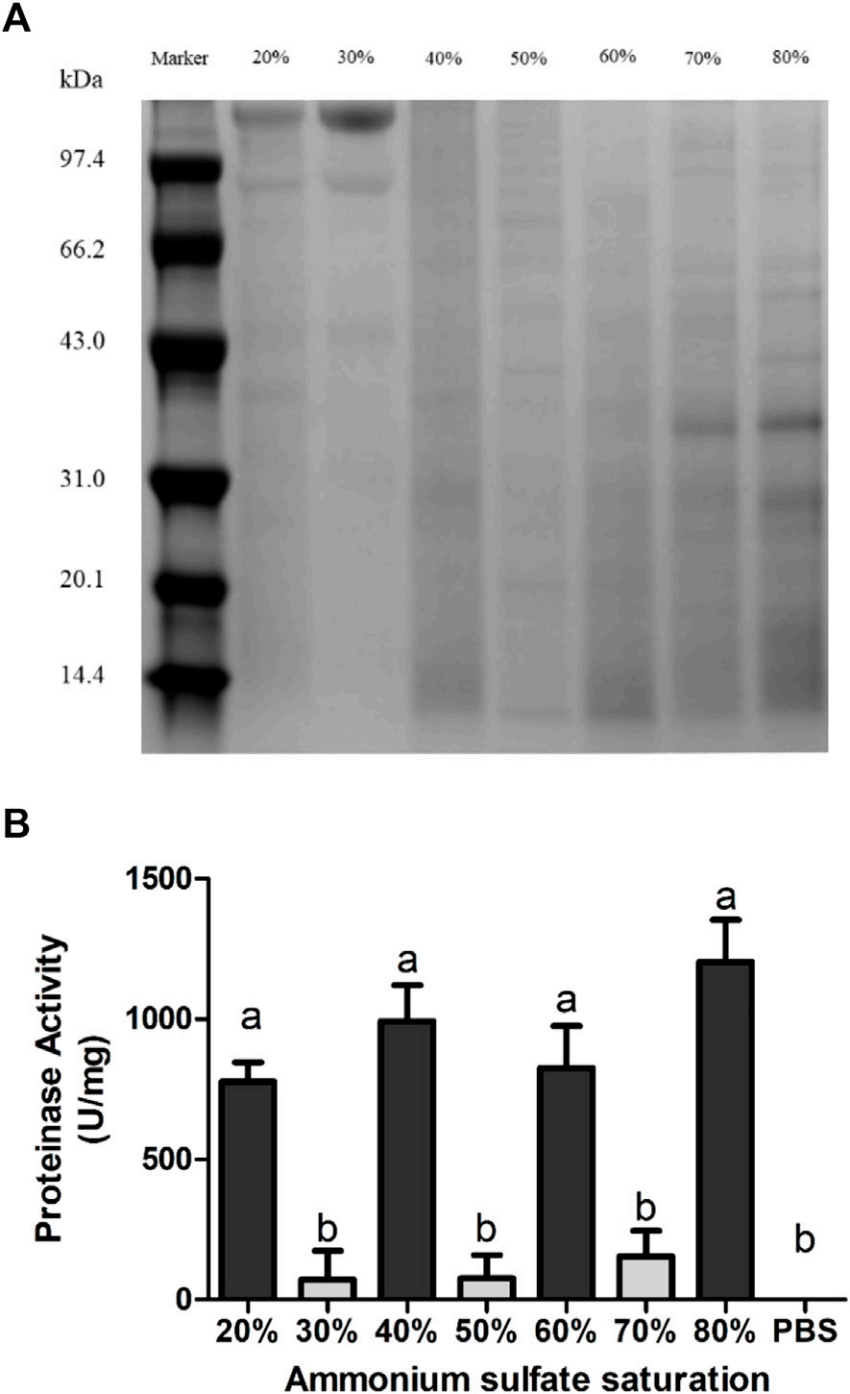
Ammonium sulfate purification of venom from NnTNV. **(A)** Nonreducing SDS-PAGE of protein fractions in different ammonium sulfate saturations. The number and location of bands of each fraction were obviously different. Bands of fraction 80% were more clear than others. **(B)** Proteinase activity of different protein fractions. Four fractions showed significant proteinase activity, *p* < 0.05. Fraction 80% showed the highest activity.

The first fractionation step, ammonium sulfate fractional precipitation, separated the venomous sample into seven fractions by 20, 30, 40, 50, 60, 70, and 80% saturation. The protein concentration of each fraction is shown in Supplementary Table S2. According to SDS-PAGE, shown in Figure 2A, proteins in NnTNV could be preliminarily separated by solubility. Several proteins, such as the proteins above 97.4 kDa molecular weight in 20 and 30% saturation and proteins between 31.0 and 43.0 kDa molecular weight in 70 and 80% saturation were separated effectively. The proteins separated by 20, 40, 60, and 80% saturation showed significant proteinase activity with similar intensity (Figure 2B). It indicated that there were at least four different proteases contained in NnTNV. The fraction separated by 80% saturation showed the highest proteinase activity measured as 1,204 U/mg in mean value and more clear electrophoretic bands than other fractions, so it was selected for the next step. The fractions separated by 30, 50, and 70% saturation did not show proteinase activity (Figure 2B).

**FIGURE 3:**
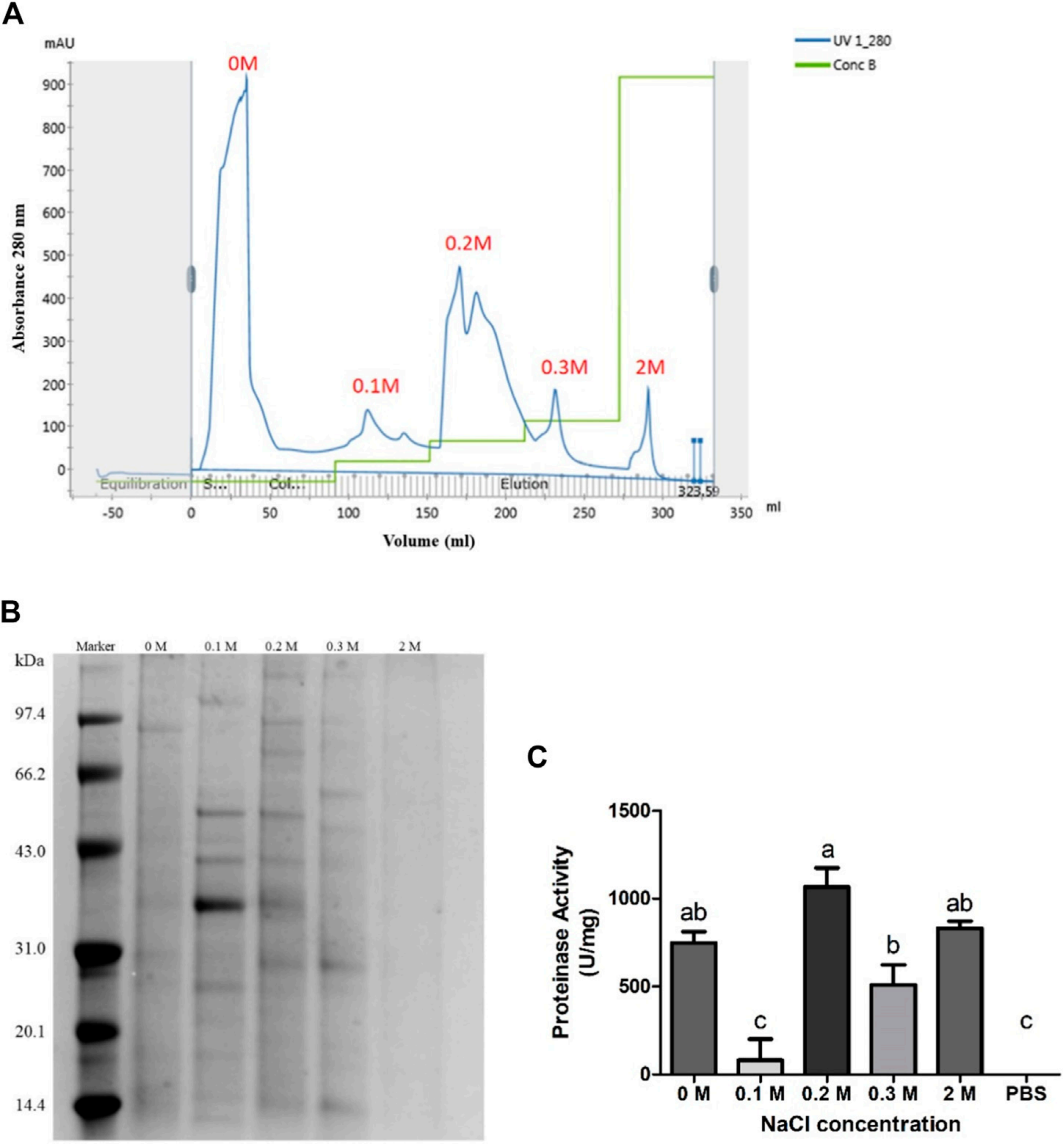
DEAE Sepharose Fast Flow Chromatography purification of protein fraction in 80% ammonium sulfate saturation. **(A)** Elution peaks of protein fraction in 80% ammonium sulfate saturation purified by DEAE Sepharose Fast Flow chromatography. Five independent peaks were obtained. **(B)** Nonreducing SDS-PAGE of each fraction. Fraction 0.1 and 0.2 M showed more clear protein bands. **(C)** Proteinase activity of each fraction. Four fractions showed significant proteinase activity, *p* < 0.05. Fraction 0.2 M showed the highest activity.

The second fractionation step was DEAE Sepharose Fast Flow chromatography. About 30 mg protein precipitation collected from 80% ammonium sulfate saturation was used in DEAE Sepharose Fast Flow chromatography. Elution peaks are shown in Figure 3A. The 80% saturation fraction was separated effectively by different ion intensities. Fractions eluted by 0 and 0.2 M NaCl contain more proteins judged by the peak shape. Fractions eluted by 0.1 and 0.2 M NaCl showed more clear electrophoretic bands (Figure 3B). The fraction eluted by 2 M NaCl did not show clear protein bands. Only the fraction eluted by 0.1 M NaCl did not show significant proteinase activity. Fractions eluted by 0 and 2 M NaCl showed similar activity intensity. The fraction eluted by 0.2 M NaCl showed the highest proteinase activity measured as 1065 U/mg, so it was selected for the next step. The protein content of this fraction was about 17.68 mg by calculating the collection volume (Figure 3A) and concentration (Supplementary Table S3). The ion intensity and proteinase activity did not show an obvious relationship (Figure 3C).

**FIGURE 4:**
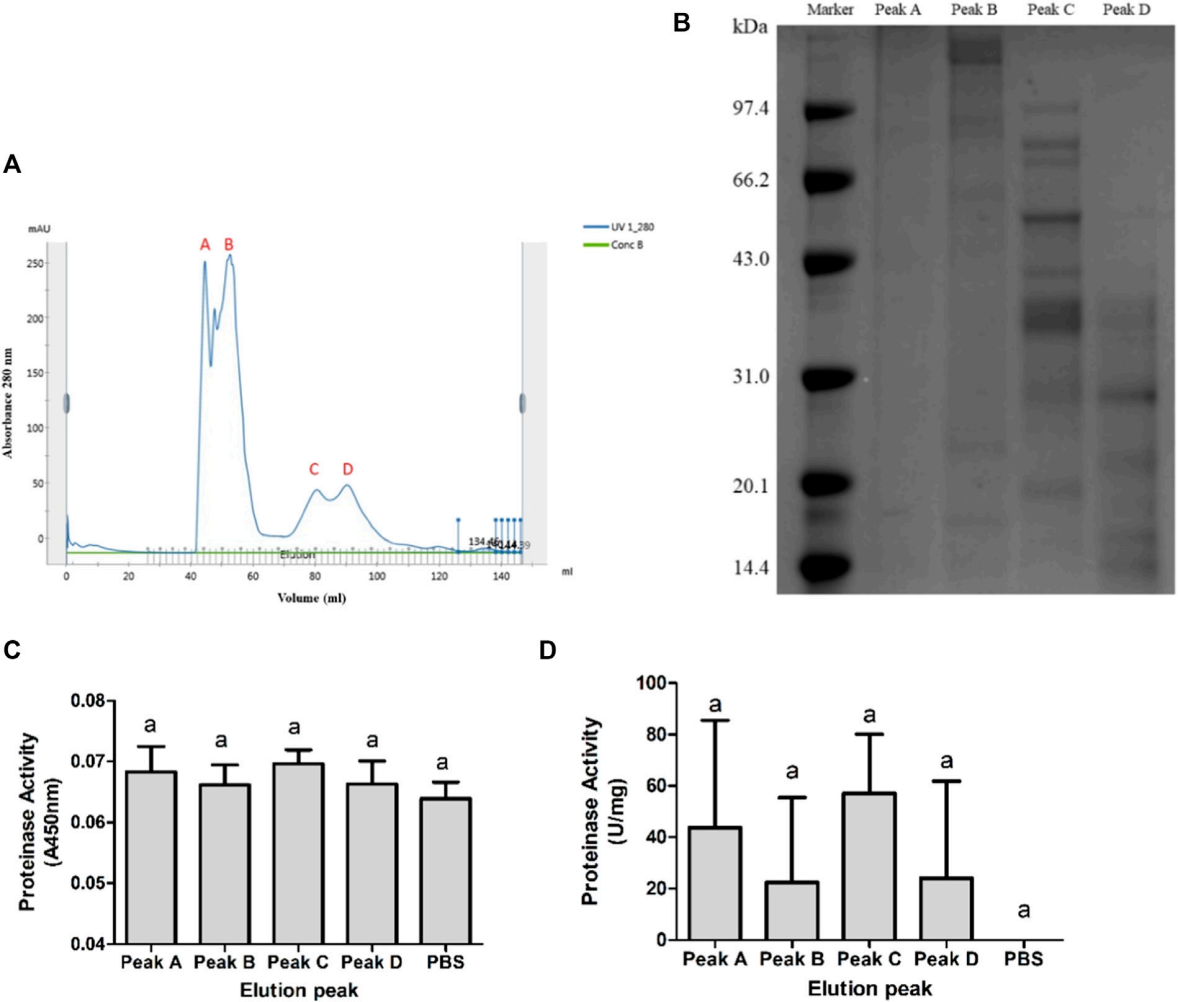
Superdex chromatography purification of 0.2 M NaCl–eluted fraction. **(A)** Elution peaks of 0.2 M NaCl–eluted fraction purified by Superdex 75. Peaks A and B were well separated from C and D. But components in AB and CD were not entirely separated. **(B)** Nonreducing SDS-PAGE of each fraction. Protein bands of C and D were similar and more clear than those of A and B. **(C,D)** Proteinase activity of each fraction showed by A450 nm and U/mg. None of the four fractions showed significant proteinase activity, *p* < 0.05.

The third fractionation step was Superdex chromatography. All collected elution sample of 0.2 M fraction was concentrated, filtered, and submitted onto a HiLoad 16/60 Superdex 75 column. The fraction eluted by 0.2 M NaCl was separated into four elution peaks as shown in Figure 4A. Elution peaks had mainly separated into peak A–B and peak C–D. Eluent was pooled into fractions A, B, C, and D. Calculating by collection volume (Figure 4A) and concentration (Supplementary Table S4), the protein content was 0.14 mg in A, 1.08 mg in B, 1.12 mg in C, and 1.63 mg in D. According to the SDS-PAGE, each fraction showed different electrophoretic bands distribution (Figure 4B). Fractions A and B had blurry bands above 66.2 kDa. Fraction C had clear bands between 20.1 and 97.4 kDa. Fraction D had clear bands below 43.0 kDa. Fraction B also had two blurry bands near 20.1 kDa. Although the fraction eluted by 0.2 M NaCl had the highest metalloproteinase activity in all DEAE Sepharose Fast Flow chromatography fractions, its elution peaks further separated by Superdex chromatography did not show significant proteinase activity to the negative control (PBS, 0 U/mg) as shown in Figures 4C,D.

### Recover the Proteinase Activity of Deactivated Fractions

**FIGURE 5:**
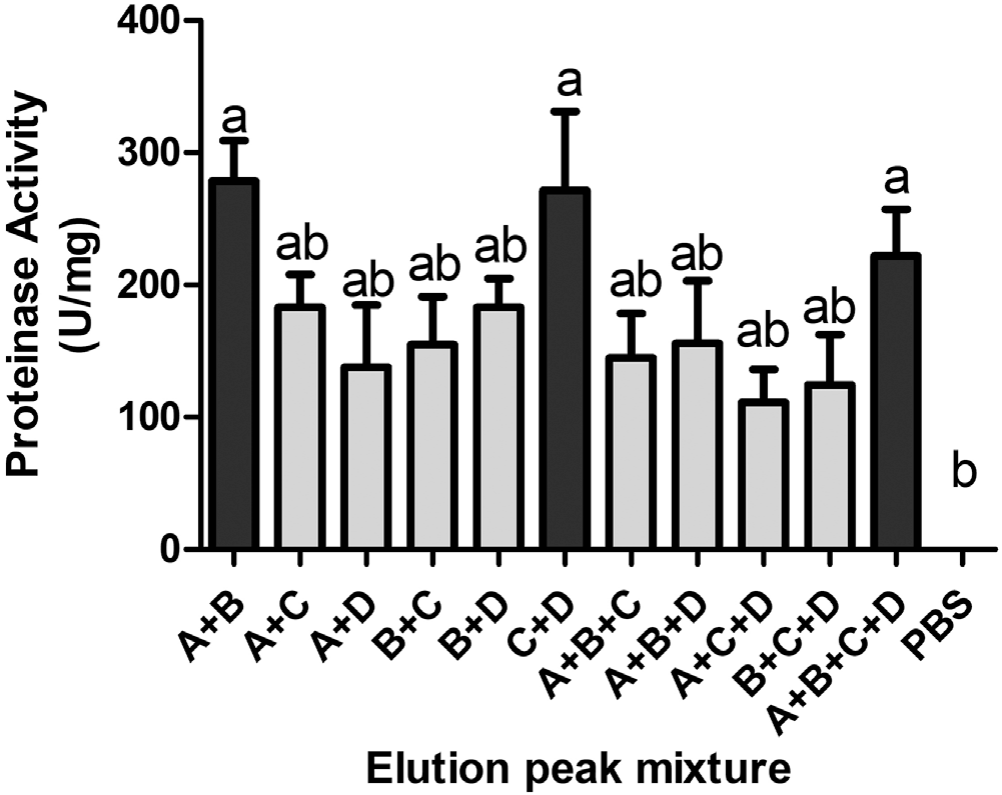
Proteinase activity of different mixtures of the four elution peaks. Each group was an equal mixture of elution peaks in the same protein content. Three groups showed significant proteinase activity, *p* < 0.05.

Deactivated fractions could recover the proteinase activity as shown in Figure 5. Mixture groups were made by mixing each fraction in equal volume and equal concentration. Three elution peak mixtures, “A + B,” “C + D,” and “A + B + C + D”, showed significant activity, measured as 220–270 U/mg, to PBS (0 U/mg). The proteinase activity of other mixtures, measured as 110–180 U/mg, did not show significant differences both to three active groups and PBS.

### LC-MS/MS Identification of Fractions C and D

According to the electrophoretic bands shown in Figure 4B, fractions C and D showed more clear bands and more obvious differences. Fractions A and B might be aggregated high molecular proteins. So, we selected fractions C and D to identify the protein components. The spectra obtained from LC-MS/MS were analyzed by the Tox-Prot database (all animal toxins database) and the *N. nomurai* genome database. The mass spectrometry proteomics data have been deposited to the ProteomeXchange Consortium *via* the PRIDE partner repository with the data set identifier PXD029333.

As seen in Tables 1, 2, the results of the Tox-Prot analysis, a total of 41 peptides in fraction C were matched to 84 proteins which were classified as 41 protein groups (Table 1), and a total of 35 peptides in fraction D were matched to 71 proteins which were classified as 34 protein groups (Table 2). Some protein groups contain many high similarity proteins. Dermonecrotic toxin in fraction D was matched by three different peptides. The matched homologous toxins both in fractions C and D were venom allergen, dermonecrotic toxin, reticulocalbin, peroxiredoxin, serine protease, cysteine-rich venom protein, venom acid phosphatase, L-amino-acid oxidase, ion channel toxin, neurotoxin, etc. Most of these shared groups were matched by the same peptides. Protein toxins only matched in fraction C were phospholipase A_1_, cystatin, zinc metalloproteinase, etc. Protein toxins only matched in fraction D were snake venom metalloprotease (SVMP) inhibitor, SVMP, hyaluronidase, etc.

**TABLE 1:** Toxins of NnTNV purification fraction C identified by LC-MS/MS analysis and Tox-Prot database.

Peptide sequence	Protein ID	Protein name	Cover percent (%)
AGATNGK	P84688	Toxin To7	8.33
	P84685	Toxin To6	8.24
ILKGGLK	C0HLS3	Delta-pseudomyrmecitoxin-Pp1a subunit B	21.21
VQIVR	A1BQQ5	Cysteine-rich venom protein Mr30	1.73
KM*LLEK	B1P1I3	U31-theraphotoxin-Cg1a	5.22
	B1P1I4	U31-theraphotoxin-Cg1b	5.22
TLQEK	B2D0J4	Venom dipeptidyl peptidase 4	0.65
LNPFR	B3EWX0	Short cationic peptide-6a	25
	B3EWX2	Short cationic peptide-6b	26.32
CLGIR	B5U2W0	Venom serine protease Bi-VSP	1.39
LICVR	B6V6L0	Conotoxin Im6.1	6.17
IVEVVK.D ! K.IVEVVK	C0HJH7	M-poneritoxin-Dq4a	21.43
	C0HJH6	M-poneritoxin-Dq4b/U1-poneritoxin-Dq4c/U1-poneritoxin-Dq4d	21.43
AAM*GTVRAK	C0HJT0	Potassium channel toxin alpha-KTx 2.19	24.32
	P0DL43	Potassium channel toxin alpha-KTx 2.14	24.32
LLM*QK	C0HLL3	Phospholipase A1	1.64
	P0CH47	Probable phospholipase A1 magnifin	1.48
EFADK	C0JB87	Dermonecrotic toxin SdSicTox-betaIIB1bxiii (Fragment)	1.82
	C0JB84	Dermonecrotic toxin SdSicTox-betaIIB1bx (Fragment)	1.82
	C0JB83	Dermonecrotic toxin SdSicTox-betaIIB1bix (Fragment)	1.82
	C0JB76	Dermonecrotic toxin SdSicTox-betaIIB1ai (Fragment)	1.82
	C0JB73	Dermonecrotic toxin SdSicTox-betaIIB1biii (Fragment)	1.82
	C0JB85	Dermonecrotic toxin SdSicTox-betaIIB1bxi (Fragment)	1.83
	C0JB80	Dermonecrotic toxin SdSicTox-betaIIB1bvi (Fragment)	1.82
	C0JB79	Dermonecrotic toxin SdSicTox-betaIIB1ai (Fragment)	1.82
	C0JB71	Dermonecrotic toxin SdSicTox-betaIIB1bi (Fragment)	1.2
	C0JB88	Dermonecrotic toxin SdSicTox-betaIIB1bxiv (Fragment)	1.83
	C0JB74	Dermonecrotic toxin SdSicTox-betaIIB1biv (Fragment)	1.82
	C0JB86	Dermonecrotic toxin SdSicTox-betaIIB1bxii (Fragment)	1.82
	C0JB82	Dermonecrotic toxin SdSicTox-betaIIB1bviii (Fragment)	1.82
	C0JB77	Dermonecrotic toxin SdSicTox-betaIIB1aii (Fragment)	1.82
	C0JB69	Dermonecrotic toxin SpaSicTox-betaIIA2 (Fragment)	1.82
	C0JB81	Dermonecrotic toxin SdSicTox-betaIIB1bvii (Fragment)	1.82
	C0JB72	Dermonecrotic toxin SdSicTox-betaIIB1bii (Fragment)	1.82
	C0JB78	Dermonecrotic toxin SdSicTox-betaIIB1aiii (Fragment)	1.83
	C0JB75	Dermonecrotic toxin SdSicTox-betaIIB1bv (Fragment)	1.82
IELTK	E3P6P2	Cystatin	3.55
	E3P6N9	Cystatin	3.55
	E3P6P0	Cystatin	3.55
	E3P6N3	AsCystatin	3.55
	E3P6N8	Cystatin	3.55
KTWSGTIIER	F8J2G5	Short neurotoxin 342	12.82
QGYISK	G0LXV8	Alpha-latrotoxin-Lh1a (Fragment)	0.44
	P23631	Alpha-latrotoxin-Lt1a	0.43
	P0DJE3	Alpha-latrotoxin-Lhe1a	0.42
IWDLK	H1ZZI8	Toxin Tpa7	6.10
IPILDGDGEATLK	I2C090	*Ophiophagus* venom factor	0.79
LSPEEQQK	J3S9D9	Reticulocalbin-2	2.61
LLDAAK	P0C1Q4	Mastoparan-1	42.86
QITMNDLPVGR	P0CV91	Peroxiredoxin-4 (Fragments)	30.56
ADLDLLR	P0DME8	Peptide Hp1239	10.45
VAACTNEIAGVK	P0DPT0	Phospholipase A1 VesT1.02	3.99
EEILR	P0DQF3	U-scoloptoxin(21)-Sm2a	7.25
EQITSRLK	P0DSJ9	U-myrmeciitoxin(01)-Mg5b	13.56
	P0DSJ8	U-myrmeciitoxin(01)-Mg5a	13.56
TRTSWDEDIMLIR	P26324	Thrombin-like enzyme ancrod	5.56
ILIHR	P43445	Short neurotoxin homolog	6.02
VSIGIK	P83108	Neurotoxin	17.14
QKNDKK	P86308	Tachykinin-like peptide-XI	46.15
ILGGIK	Q2XXP5	Cysteine-rich venom protein TEL1 (Fragment)	2.86
ESLEK	Q4JHE2	L-amino-acid oxidase	0.97
HM*LDVVSGTQK	Q5BLY5	Venom acid phosphatase Acph-1	2.84
EYLMK	Q5D7H4	Inactive hyaluronidase B	1.47
ELSIR	Q5Y4X2	U2-agatoxin-Ao1n	7.14
VLFDK	Q68Y22	M-myrmeciitoxin-Mb2a	5.95
GAEIIR	Q6XLL6	Potassium channel toxin alpha-KTx 6.9	9.84
LPNKDR	Q7SYF1	Thrombin-like enzyme cerastocytin	2.34
MELIR	Q8AY79	Beta-fibrinogenase stejnefibrase-2	1.94
	Q8AY80	Alpha- and beta-fibrinogenase stejnefibrase-1	1.94
	Q91516	Venom plasminogen activator TSV-PA	1.94
KENGRK	Q8JIR2	Zinc metalloproteinase/disintegrin-like HR1a	0.99
	Q10749	Snake venom metalloproteinase–disintegrin-like mocarhagin	0.99
	Q4VM08	Zinc metalloproteinase–disintegrin-like VLAIP-A	0.97
	Q2UXQ5	Zinc metalloproteinase–disintegrin-like EoVMP2	0.98
	Q2UXR0	Zinc metalloproteinase–disintegrin-like Eoc1	0.98
	B8K1W0	Zinc metalloproteinase–disintegrin-like daborhagin-K	0.98
	Q7T046	Coagulation factor X–activating enzyme heavy chain	0.98
	Q7LZ61	Coagulation factor X–activating enzyme heavy chain	0.97
LVPIASK	Q98956	Cytotoxin 1b	8.64
	P86538	Cytotoxin 2a	11.67
LLNKR.S ! K.LLNKR	Q9BPF2	Conotoxin Vn-05	8.20
	Q68IP5	Conotoxin mr5.4b (Fragment)	9.26
VATGK	W4VS53	CRISP/Allergen/PR-1	1.23

**TABLE 2:** Toxins of NnTNV purification fraction D identified by LC-MS/MS analysis and Tox-Prot database.

Peptide sequence	Protein ID	Protein name	Cover percent (%)
LSPEEQQK	J3S9D9	Reticulocalbin-2	4.25
QLHLK			
K.ILKGGLK.S	C0HLS3	Delta-pseudomyrmecitoxin-Pp1a subunit B	21.21
XIIGAPCRR	P0C7W7	Kappa-stichotoxin-Shd1a/kappa-stichotoxin-Shd1b	32.14
EEILR	P0DQF3	U-scoloptoxin(21)-Sm2a	7.25
RSEHEEQLMAK	P0DQF5	U-scoloptoxin(22)-Er1a	8.15
TRTSWDEDIMLIR	P26324	Thrombin-like enzyme ancrod	5.56
QASQKWGR	A8YPR6	Snake venom metalloprotease inhibitor 02D01	2.60
LNPFR	B3EWX0	Short cationic peptide-6a	25.00
	B3EWX2	Short cationic peptide-6b	26.32
SCAGMGQDCK	B6DD25	U13-lycotoxin-Ls1f	8.33
	B6DD28	U13-lycotoxin-Ls1b	8.33
	B6DD26	U13-lycotoxin-Ls1f	8.33
LDTVR	C0JB09	Dermonecrotic toxin LarSicTox-alphaIII1 (Fragment)	1.82
EFADK	C0JB80	Dermonecrotic toxin SdSicTox-betaIIB1bvi (Fragment)	1.82
	C0JB73	Dermonecrotic toxin SdSicTox-betaIIB1biii (Fragment)	1.82
	C0JB79	Dermonecrotic toxin SdSicTox-betaIIB1ai (Fragment)	1.82
	C0JB71	Dermonecrotic toxin SdSicTox-betaIIB1bi (Fragment)	1.82
	C0JB77	Dermonecrotic toxin SdSicTox-betaIIB1aii (Fragment)	1.82
	C0JB69	Dermonecrotic toxin SpaSicTox-betaIIA2 (Fragment)	1.82
	C0JB85	Dermonecrotic toxin SdSicTox-betaIIB1bxi (Fragment)	1.83
	C0JB88	Dermonecrotic toxin SdSicTox-betaIIB1bxiv (Fragment)	1.83
	C0JB84	Dermonecrotic toxin SdSicTox-betaIIB1bx (Fragment)	1.82
	C0JB83	Dermonecrotic toxin SdSicTox-betaIIB1bix (Fragment)	1.82
	C0JB87	Dermonecrotic toxin SdSicTox-betaIIB1bxiii (Fragment)	1.82
	C0JB76	Dermonecrotic toxin SdSicTox-betaIIB1ai (Fragment)	1.82
	C0JB86	Dermonecrotic toxin SdSicTox-betaIIB1bxii (Fragment)	1.82
	C0JB78	Dermonecrotic toxin SdSicTox-betaIIB1aiii (Fragment)	1.83
	C0JB82	Dermonecrotic toxin SdSicTox-betaIIB1bviii (Fragment)	1.82
	C0JB74	Dermonecrotic toxin SdSicTox-betaIIB1biv (Fragment)	1.82
	C0JB81	Dermonecrotic toxin SdSicTox-betaIIB1bvii (Fragment)	1.82
	C0JB75	Dermonecrotic toxin SdSicTox-betaIIB1bv (Fragment)	1.82
	C0JB72	Dermonecrotic toxin SdSicTox-betaIIB1bii (Fragment)	1.82
LTEALK	C0JB90	Dermonecrotic toxin SdSicTox-betaIIB2ii (Fragment)	2.19
	C0JB89	Dermonecrotic toxin SdSicTox-betaIIB2i (Fragment)	2.19
	C0JB91	Dermonecrotic toxin SaSicTox-betaIIB1 (Fragment)	2.19
KLDLR	D2Y2H8	U6-theraphotoxin-Hhn1a 4	5.15
	D2Y2H6	U6-theraphotoxin-Hhn1a 2	5.15
	D2Y2C1	U6-theraphotoxin-Hhn1a 1	5.15
	D2Y2H7	U6-theraphotoxin-Hhn1a 3	5.15
MIIFK	G3LU44	Translationally controlled tumor protein homolog	2.91
	M5B4R7	Translationally controlled tumor protein homolog	2.89
IWDLK	H1ZZI8	Toxin Tpa7	6.10
TGVEIK	P01393	Alpha-elapitoxin-Djk2a	8.33
QITMNDLPVGR	P0CV91	Peroxiredoxin-4 (Fragments)	30.56
ADLDLLR	P0DME8	Peptide Hp1239	10.45
EQITSRLK	P0DSJ8	U-myrmeciitoxin(01)-Mg5a	13.56
	P0DSJ9	U-myrmeciitoxin(01)-Mg5b	13.56
GLPEDAK	P0DUI0	Beta-toxin Ct13	8.64
NEILK	P10736	Venom allergen 5.01	2.20
LEILK	P81657	Venom allergen 5	2.48
	P0DMB9	Venom allergen 5	2.48
	P86870	Venom allergen 5	2.22
LVPIASK	P86538	Cytotoxin 2a	11.67
	Q98956	Cytotoxin 1b	8.64
ILGGIK	Q2XXP5	Cysteine-rich venom protein TEL1 (Fragment)	2.86
QEYGAERLR	Q3YEG6	Conotoxin LiC42	11.69
YENFNDFLK	Q4VDB5	Dermonecrotic toxin LgSicTox-alphaIA1	3.21
	P0CE80	Dermonecrotic toxin LiSicTox-alphaIA1a	2.94
	Q56JA9	Dermonecrotic toxin LsSicTox-alphaIA1	3.21
	P0CE82	Dermonecrotic toxin LiSicTox-alphaIA1bii (Fragment)	2.98
	P0CE81	Dermonecrotic toxin LiSicTox-alphaIA1bi	2.94
LNLIR	Q5BLY5	Venom acid phosphatase Acph-1	1.29
KVHEVK	Q6T627	L-amino-acid oxidase (Fragment)	10.00
GAEVIR	Q6XLL5	Potassium channel toxin alpha-KTx 6.10	10.00
GAEIIR	Q6XLL6	Potassium channel toxin alpha-KTx 6.9	9.84
TIEELAK	Q75WG7	U13-hexatoxin-Mg1a	5.65
LENVEKEDGGPK	Q8JJ51	Snake venom metalloproteinase	2.90
LLNKR.S ! K.LLNKR	Q9BPF2	Conotoxin Vn-05	8.20
	Q68IP5	Conotoxin mr5.4b (Fragment)	9.26
DILDK.S ! K.DLLDK	R4J7Z9	Hyaluronidase	1.25
	F8J2D3	Phospholipase-B 81	0.90

The result of *N. nomurai* genome database analysis is shown in Table 3. Only two mitochondrion proteins, ATP synthase F0 subunit 8 and NADH dehydrogenase subunit 4L, were matched in fraction C.

**TABLE 3:** Proteins of NnTNV purification fraction C identified by LC-MS/MS analysis and *N. nomurai* genome database.

Peptide sequence	CDS	Gene description	Cover percent (%)
LNEVR	YP_009421312.1	ATP synthase F0 subunit 8	7.46
LLNILK	YP_009421319.1	NADH dehydrogenase subunit 4 L	7.23

## Discussion

As jellyfish stings pose a threat to humans in many marine activities in summer, especially swimming, the study of jellyfish sting mechanism is necessary for therapy researches. *N. nomurai* is a giant jellyfish widely distributed in the Yellow Sea and East China Sea, which blooms in summer in recent years (Sun et al., 2015a; Sun et al., 2015b). In *N. nomurai* venom, metalloproteinase is a major component (Li et al., 2014). NnVMPs showed azocasein hydrolysis activity and impacted the expression of many inflammatory factors (Li et al., 2019) and increased vascular permeability by directly degrading basement membrane components (Yue et al., 2021).

Metalloproteinases widely exist in venomous animals, including snakes, scorpions, spiders, jellyfish, etc. and play an important role in digestion and preying. Many different SVMPs are obtained by two or three steps of chromatography, such as CCSV-MPase (Cherifi et al., 2010), Atrase B (Sun and Bao, 2010), BmooMP α -II (de Queiroz et al., 2014), and CcD-II (Ami et al., 2017). Additionally, some metalloproteinases can be obtained by cDNA cloning and expression, such as rFIVa (Xu et al., 2006), Ahpfibrase (Zhang et al., 2010), and Jerdonitin (Zhu et al., 2010). Owing to the successful single protein purification, many SVMPs were studied in-depth in structures, functions, and mechanisms. SVMPs can be classified by their domain architecture into type P-I to P-III (Fox and Serrano, 2008). SVMPs are multifunction proteins, and they showed hemorrhagic, procoagulant, anticoagulant, and antiplatelet effects in envenomation cases (Fox and Serrano, 2005). Researches on the jellyfish venom purification are relatively difficult. Only a few toxins were highly purified from jellyfish venom, such as cytotoxin ClGp1, cytotoxin CcTX-1, neurotoxin CcNT, antioxidant protein SmP90 (Rottini et al., 1995; Yang et al., 2003; Helmholz et al., 2008; Lassen et al., 2011; Lassen et al., 2012; Li et al., 2012; Horiike et al., 2015), etc. Techniques most used in venom protein purification were activity-guided multidimensional chromatography, including size-exclude, ion-exchange, reversed-phase, and affinity chromatography. Lassen purified cytotoxin CcTX-1 and neurotoxin CcNT from *Cyanea capillata* in this program (Lassen et al., 2011; Lassen et al., 2012). Nevertheless, most jellyfish protein toxins could only be partially purified by multi-chromatography, such as a 95 kDa metalloproteinase from *R. pulmo* (barrel jellyfish) venom (Rastogi et al., 2017) and other hemolytic and lethal jellyfish toxins, such as SmTX from *S. meleagris* (Li et al., 2013) and NnLF from *N. nomurai* (Li et al., 2020). Due to the complex composition, it is not easy to get a single toxin component from jellyfish venom. In jellyfish venom, some different protein components have very similar physical properties such as molecule weight. In addition, toxins in jellyfish venom contained different subfamilies and may interact with other components. It was hard to efficiently isolate a single jellyfish toxin protein within two or three chromatography steps. In addition, the protein content in jellyfish nematocyst venom is too small an amount to do repeated chromatography, which makes it difficult to reveal the biological activity of a single toxin component. In this study, proteases from NnTNV were purified by ammonium sulfate precipitation, DEAE Sepharose Fast Flow, and Superdex 75 column chromatography successively. During the purification process, the proteinase activity of 80% saturation fraction and 0.2 M ion intensity fraction was increased from 314 U/mg to 1,204 U/mg and 1,065 U/mg, respectively. But none of the final fractions in Superdex chromatography showed significant proteinase activity to PBS (Figures 4C,D). However, when these fractions were mixed again by the same protein content, the mixed samples “A + B,” “C + D,” and “A + B + C + D” recovered the activity (Figure 5), which indicated that there was synergistic effect between the final fractions.

Synergistic effect is common in animal venom such as many snake toxins (Doley and Kini, 2009; Xiong and Huang, 2018). Currently, there is no universal method to study the synergistic effects of animal toxins. Laustsen proposed the Toxicity Score method to determine the presence of synergism in venom (Lauridsen et al., 2016). Venom synergistic effect is mainly revealed by the research of venom purification and molecular mechanisms. For example, SVMP may interact with phospholipase A_2_ (Xiong et al., 2017). The enzyme activity of P-III metalloproteinase complex was regulated by its subunits (Doley and Kini, 2009). Although there has been no research in jellyfish metalloproteinase synergistic effect, unrevealed interaction of synergistic proteins may impact the bioactivity and purification studies.

As the final fractions were multicomponent, the protein components of fractions C and D were identified by LC-MS/MS to reveal the possible mechanism of proteinase activity regulation in *N. nomurai* venom. However, few jellyfish toxins are well understood. A large number of protein toxins were matched in snakes, spiders, scorpions, bees, and other organisms (Table 1, Table 2) by searching in Tox-Prot. The unique peptide count of most matched proteins was only one, but some peptides matched many similar proteins in different organisms, such as peptide KENGRK which was matched to eight different zinc metalloproteinases. It made the peptide sequence more probable to represent the matched protein group. This means even the unique peptide count and cover percent were not high, these matched toxins from other organisms also provided very important references. Similar to the chromatography and SDS-PAGE results, toxins matched in fractions C and D showing shared and different components. These different toxins contained in fractions C and D might contain the regulator of proteinase activity, such as phospholipase A1, P-III metalloproteinase, P-I metalloproteinase, and metalloproteinase inhibitor.

In fraction C, the matched toxin proteins which related to proteinase activity were zinc metalloproteinase and serine protease. The peptide sequence matched to zinc metalloproteinase was KENGRK. It matched eight similar P-III metalloproteinases from different snakes. They can produce proteolytic activity and hemorrhage activity (Howes et al., 2003; Morine et al., 2008). This kind of metalloproteinase has metalloproteinase domain, disintegrin domain, and cysteine-rich domain (Gowda et al., 1994; Siigur et al., 2001; Siigur et al., 2004; Trummal et al., 2005; Takeda et al., 2007; Chen et al., 2008). Sequence KENGRK is located in the cysteine-rich domain. The peptide sequences matched to serine protease were CLGIR, TRTSWDEDIMLIR, and LPNKDR. CLGIR matched venom serine protease Bi-VSP from *Bombus ignitus* (bumblebee). TRTSWDEDIMLIR matched thrombin-like enzyme ancrod from *Calloselasma rhodostoma* (Malayan pit viper). LPNKDR matched thrombin-like enzyme cerastocytin from *Cerastes cerastes* (horned desert viper). These serine proteases have fibrinogenolytic activity (Marrakchi et al., 1995; Marrakchi et al., 1997; Dekhil et al., 2003; Choo et al., 2010; Choo et al., 2012).

In fraction D, the matched toxin proteins which related to proteinase activity were SVMP, SVMP inhibitor, and serine protease. Serine protease was matched by peptide sequence TRTSWDEDIMLIR which was also identified in fraction C. The sequence LENVEKEDGGPK was matched to SVMP from *Crotalus molossus molossus* (Northern black-tailed rattlesnake). This kind of P-I metalloproteinase impairs hemostasis in the envenomed animal (Gutierrez et al., 1995; Farsky et al., 2000; Patino et al., 2010; Bernardes et al., 2013). The sequence QASQKWGR was matched to SVMP inhibitor 02D01 from *Echis ocellatus* (ocellated saw-scaled viper). This protein may inhibit metalloproteinase activity in the venom gland through abundant pEKW and poly-His-poly-Gly peptides. The inhibition may be disengaged by dilution or physiochemical change (Wagstaff et al., 2008).

These SVMP-like proteins in fractions C and D might be the important factors in regulating azocasein hydrolysis activity. Zinc metalloproteinases matched in fraction C were P-III metalloproteinase which contains metalloproteinase domain, disintegrin domain, and cysteine-rich domain (Gowda et al., 1994; Siigur et al., 2001; Siigur et al., 2004; Trummal et al., 2005; Takeda et al., 2007; Chen et al., 2008). Some of this kind of P-III metalloproteinase were confirmed that they can constitute complexes with C-type lectin homodimers light chains, such as RVV-X (Gowda et al., 1994; Takeda et al., 2007) and CA-1 (Yamada et al., 1996). RVV-X is a P-III metalloproteinase complex isolated from *Daboia siamensis* (Eastern Russel’s viper) which can activate coagulation factor X by cleavage of Arg-|-Xaa bonds (Gowda et al., 1994; Takeda et al., 2007). The heavy chain (Protein ID, Q7LZ61) was matched in fraction C. In RVV-X complex, the heavy chain is the catalytic subunit of activating coagulation factor X, and the two light chains are regulatory subunits of binding the Gla domain of factor X (Takeya et al., 1992). Similar to RVV-X, this protein group can selectively hydrolyze factor X to Xa, but some of them were also confirmed having azocasein hydrolysis activity such as the protein zinc metalloproteinase–disintegrin-like bothropasin from *Bothrops jararaca* (Mandelbaum et al., 1982). However, the zinc metalloproteinase–disintegrin-like atrase B isolated from *Naja atra* (Chinese cobra) did not show hydrolysis activity to fibrin, azocasein, and BAEE (Cherifi et al., 2010). Therefore, it was speculated that a zinc metalloproteinase which can hydrolyze azocasein was contained in fraction C, and its activity could be activated by binding with regulatory subunits contained in fraction D. Differences from the C-type lectin subunits of RVV-X, the regulatory subunits in fraction D, might affect the active center of the zinc metalloproteinase, similar to allosteric regulation. According to Table 2, fraction D might contain similar proteins to P-I SVMP and SVMP inhibitor. This indicated that there might have been an interaction between them that could inhibit the metalloproteinase activity. The inhibition of SVMP inhibitor may be disengaged by dilution or physiochemical change (Wagstaff et al., 2008).

**FIGURE 6:**
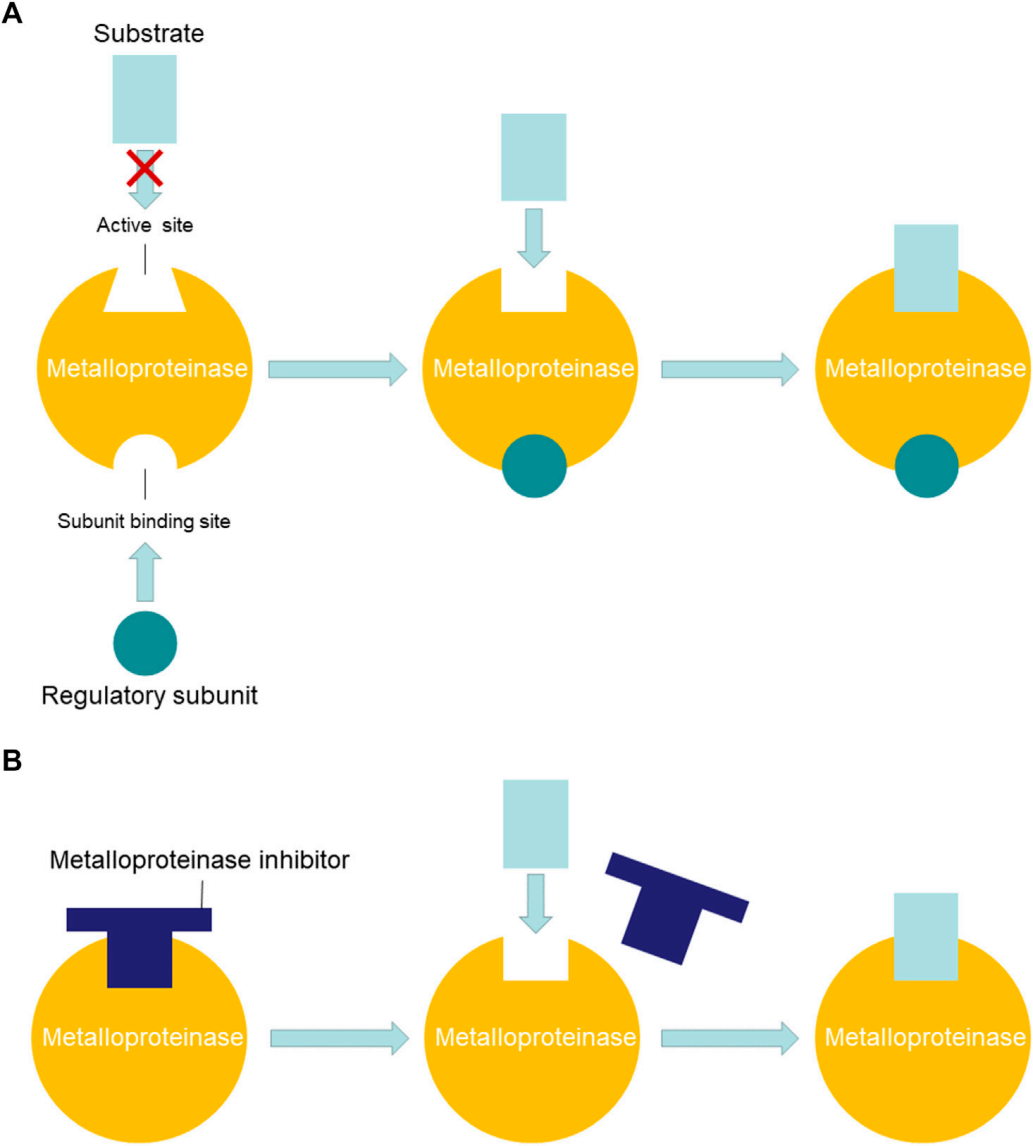
Possible regulation mechanisms of NnVMP activity. **(A)** Metalloproteinase was activated by binding regulatory subunit. **(B)** Metalloproteinase was activated by disengaging inhibitor.

To sum up the regulation mechanism, inactivated fractions C and D would recover the metalloproteinase activity by binding regulator or disaggregating inhibitor. Fraction C contained a zinc metalloproteinase which could hydrolysis azocasein under the activation of regulatory subunits in fraction D (Figure 6A). In another explanation, the interacted metalloproteinase and inhibitor in fraction D would disengage to activate metalloproteinase activity when fractions C and D were mixed (Figure 6B).

**FIGURE 7:**
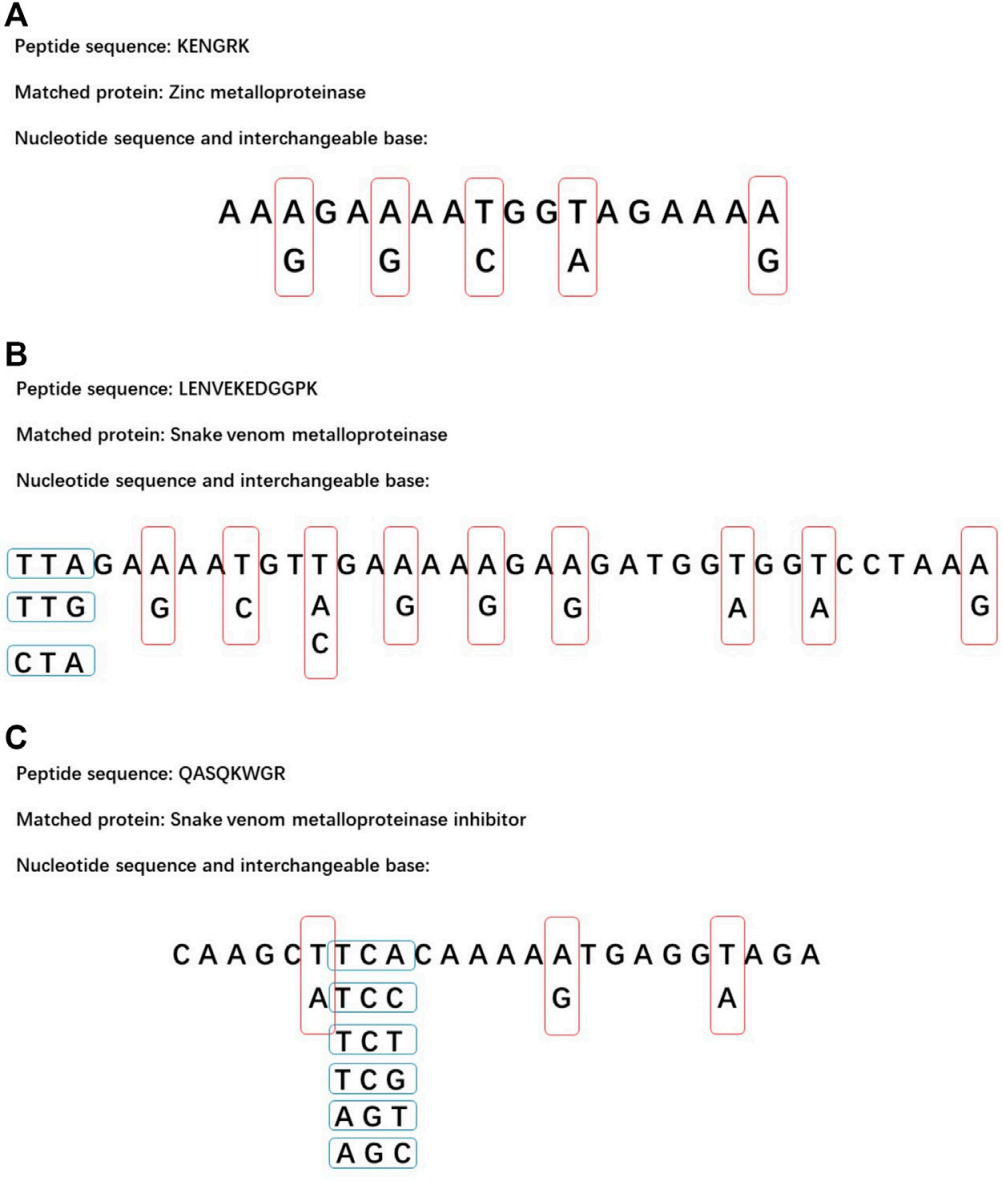
Possible nucleotide sequence of three important peptides. The red and blue frames represent interchangeable bases. **(A)** The sequence of KENGRK. **(B)** The sequence of LENVEKEDGGPK. **(C)** The sequence of QASQKWGR.

The spectra in *N. nomurai* genome database were also searched. The whole-genome database was uploaded to NCBI by Hak-Min Kim's team (Kim et al., 2019). LC-MS/MS analysis did not match any toxins in this database. BLAST of SVMP, which was matched in fractions C and D, also had no significant similarity found. Although the nucleotide sequences of *N. nomurai* toxins were not obtained, some important peptide sequences were transformed into nucleotide sequence. The nucleotide sequence of 21 amino acids shown in Table 4 was obtained from the two *N. nomurai* mitochondrion proteins listed in Table 3. Through the comparison of the protein sequence and nucleotide sequence of ATP synthase F0 subunit 8 and NADH dehydrogenase subunit 4 L in *N. nomurai*, two interesting differences were found. In *N. nomurai*, the isoleucine (Ile, I) codon AUA would translate to methionine (Met, M), and the termination codon UGA would translate to tryptophan (Try, W). This should be paid special attention in further expression works. Based on this nucleotide sequence table, three peptides, which were matched to zinc metalloproteinase, SVMP, and SVMP inhibitor, were transformed into nucleotide sequence as shown in Figure 7. These sequences may provide references for further studies interested in NnVMP.

**TABLE 4:** The nucleotide sequence of 21 amino acids in *Nemopilema nomurai*.

Amino acid	DNA sequence	mRNA sequence (codon)
M	ATG/ATA	AUG/AUA (I)
P	CCT	CCU
Q	CAA	CAA
L	TTA/TTG/CTA	UUA/UUG/CUA
D	GAT	GAU
I	ATT/ATA/ATC	AUU/AUA/AUC
V	GTT/GTA/GTC	GUU/GUA/GUC
T	ACA/ACT	ACA/ACU
F	TTT/TTC	UUU/UUC
N	AAT/AAC	AAU/AAC
Y	TAT/TAC	UAU/UAC
W	TGA	UGA (termination codon)
G	GGT/GGA	GGU/GGA
S	TCA/TCC/TCT/TCG/AGT/AGC	UCA/UCC/UCU/UCG/AGU/AGC
K	AAA/AAG	AAA/AAG
E	GAA/GAG	GAA/GAG
R	AGA	AGA
H	CAT	CAU
A	GCT/GCA	GCU/GCA
T	ACA	ACA
C	TGT	UGU

It has been a problem for a long time that jellyfish venom purification has been limited by the complex protein components. As shown in each purification step, different proteins may have similar physical properties and are hard to be completely isolated by chromatography. Low protein content also limit sample reloading of chromatography. As a new revealed property of *N. nomurai* toxins, the synergistic effect of proteinase activity may impact further activity-guided chromatography studies. Although jellyfish venom purification is difficult, it might reveal more venom properties in the purification process and provide references for further studies.

In conclusion, the toxic protease components from NnTNV were isolated by ammonium sulfate precipitation, DEAE Sepharose Fast Flow, and Superdex 75 column chromatography successively. After the three purification steps, the main proteinase activity was lost, but it could be recovered by mixing again. This is the first time that the synergistic effect of jellyfish proteinase activity has been revealed. Through LC-MS/MS analysis, it has been shown that the proteinase activity might have been contributed by metalloproteinases and regulated by metalloproteinase subunit or metalloproteinase inhibitor. Three important peptide sequences were transformed into nucleotide sequences to provide more information on *N. nomurai* metalloproteinases. The results could help further research in jellyfish toxins purification and expression. The synergistic effect might be a new entry point in purifying *N. nomurai* metalloproteinases and regulators.

## Data Availability

The data sets presented in this study can be found in online repositories. The names of the repository/repositories and accession number(s) can be found below: ProteomeXchange with identifier PXD029333.
