# Supplementary material for: Synergistic Effect of Proteinase Activity by Purification and Identification of Toxic Protease From Nemopilema nomurai
Source: Front Pharmacol. 2021 Nov 25;12:791847. doi: 10.3389/fphar.2021.791847 (PMC8660593; doi:10.3389/fphar.2021.791847)
Supplement: Supplementary file 1 [file DataSheet1.docx]

Supplementary Material

## Supplementary Tables

**Supplementary Table S1:** Comparison of sample information of NnNV and NnTNV

|  | **Sample volumes**  **(ml)** | **Protein concentration**  **(mg/ml)** | **Proteinase activity**  **(U/mg)** | **Total proteinase activity**  **(U)** |
| --- | --- | --- | --- | --- |
| **NnNV** | **145** | **1.14** | **438.6** | **7.25×10^4^** |
| **NnTNV** | **3000** | **2.84** | **314.4** | **2.68×10^6^** |

**Supplementary Table S2**: Protein concentration of ammonium sulfate precipitation fractions

| Saturation  (%) | 20 | 30 | 40 | 50 | 60 | 70 | 80 |
| --- | --- | --- | --- | --- | --- | --- | --- |
| Concentration  (µg/mL) | 186.34 | 251.06 | 78.47 | 657.89 | 405.16 | 753.43 | 207.91 |

**Supplementary Table S3**:Protein concentration of DEAE chromatography fractions

| NaCl concentration  (M) | 0 | 0.1 | 0.2 | 0.3 | 2 |
| --- | --- | --- | --- | --- | --- |
| Concentration  (µg/mL) | 236.18 | 91.94 | 265.77 | 58.65 | 6.88 |

**Supplementary Table S4**: Protein concentration of Superdex chromatography fractions

| Elution peak | A | B | C | D |
| --- | --- | --- | --- | --- |
| Concentration  (µg/mL) | 34.27 | 67.55 | 70.01 | 42.88 |
